# Heat-induced modifications of pea protein: Implications for solubility and digestion behaviour

**DOI:** 10.1016/j.crfs.2025.101173

**Published:** 2025-08-16

**Authors:** Dan Li, Ying Ma, Alejandra Acevedo-Fani, Weihong Lu, Harjinder Singh, Aiqian Ye

**Affiliations:** a Riddet Institute, Massey University, Private Bag 11 222, Palmerston North, 4442, New Zealand; b School of Chemistry and Chemical Engineering, Harbin Institute of Technology, Harbin, 150001, China

**Keywords:** Pea protein isolate, Heat treatment, Protein solubility, Hydrolysis, Gastric emptying, Protein digestibility

## Abstract

Plant proteins have become increasingly desirable due to their sustainability and proposed health benefits. This study initially examined the effects of heat treatment on the solubility of pea protein (PP) in a 3 % (w/w) protein solution, applying heat from 65 °C to 95 °C for varying durations across pH conditions ranging from 5.5 to 7.8. Subsequently, an advanced dynamic gastric digestion model—the Human Gastric Simulator—was employed to examine the *in vitro* gastric digestion behaviours of heat-treated and untreated PP. Results suggest that heat treatment reduces the protein aggregate size and enhances PP solubility, potentially due to a decrease in α-helix and β-turn structures or an increase in β-sheet content, as determined via Fourier transform infrared spectroscopy. Additionally, heat treatment elevated the surface hydrophobicity and free sulfhydryl group concentration of PP. During *in vitro* dynamic gastric digestion with pepsin, PP underwent notable structural and physical stability modifications. Unheated and heated PP exhibited small particles in the digesta and remained unaggregated throughout digestion. However, the heat-treated PP showed a smaller particle size during gastric digestion and a greater hydrolysis rate than the unheated protein. This study systematically evaluates the solubility and digestion behaviour of PP subjected to food processing conditions, highlighting its stability and structural changes that may influence the delivery of macronutrients from the stomach to the next phase of digestion.

## Highlights

• Systematic investigation revealed how heat and pH modulate pea protein structure and solubility.
• Dynamic gastric digestion uncovered distinct digestion behaviors between native and heated pea proteins.
• Heating at pH > 7 improved the solubility of pea protein and reduced protein aggregate size.
• Heated pea protein showed a smaller particle size and greater gastric digestion rate.

## Graphical abstract

**Figure:**
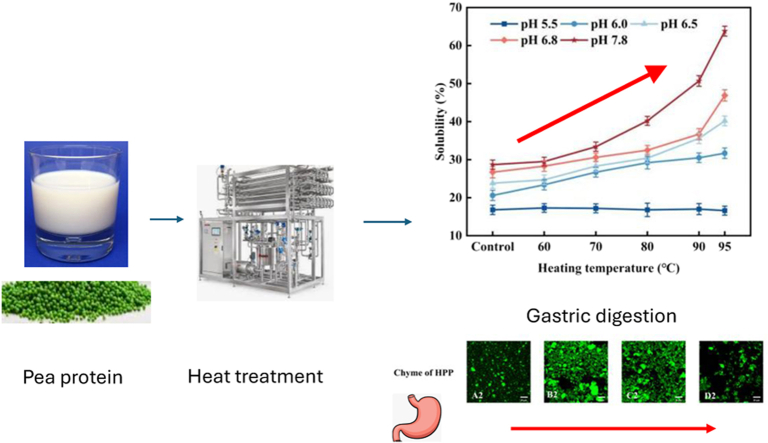

## 1 Introduction

Pea protein (PP) has gained significant attention in plant-based food development (Taylor et al., 2021). The amino acid profile of PP is well-balanced, with levels of the other seven essential amino acids, except methionine, close to the FAO/WHO recommended pattern values (Schneider et al., 2000; Spaccasassi et al., 2024). Compared with other plant proteins, PP exhibits distinct nutritional properties, a low hypoallergenic risk and high antioxidant potential (Oliete et al., 2018). PP can be classified into four types: globulin (55 %–65 %), albumin (18 %–25 %), prolamin (4 %–5 %) and glutelin (3 %–4 %). Globulins are divided into legumin (11S) and pea globulins (7S), with a 7S-to-11S globulin ratio ranging from 0.5 to 1.7, averaging around 1.1 (Spaccasassi et al., 2024; Schroeder et al., 1982). Globulins demonstrate limited solubility in aqueous media due to their spatial structures, particularly in acidic beverages, potentially impacting the formulation and texture of food products (Xiong et al., 2018).

To enhance the solubility of plant proteins, researchers have explored chemical, physical and biological treatments (Nikbakht, Sedaghat and Mezzenga, 2021). Among these, heat treatment is regarded as one of the most widely used methods for modifying protein structures. Heating causes proteins to unfold, exposing previously buried hydrophobic groups on the surface. This process alters protein solubility and surface hydrophobicity (Wang et al., 2012). Additionally, heat treatment can influence functional properties such as emulsifying capacity, foaming ability and water-binding capacity. These effects depend on specific heat treatment conditions, including temperature and exposure time (Peng et al., 2016). According to Peng et al. (2016), thermal treatment of PP at 95 °C for 30 min increased protein aggregation, while the hydrodynamic diameter grew as the concentration of heated protein increased. Asen et al. (2022) examined the effect of thermal pre-treatment of PP at different pH levels on the formation of functional protein aggregates. They found that pH levels of 3, 7 and 9 significantly influenced heat treatment, promoting more flexible protein aggregate arrangements and network formation. Beck et al. (2017) found that heat treatment of PP in the capillary rheometer resulted in protein aggregates, likely comprising β-turn structures or anti-parallel β-sheets. Heat treatment may affect the digestion behaviour of proteins (Loveday et al., 2023). However, limited information is available on the physicochemical behaviour of plant proteins in gastrointestinal environments, particularly concerning PP.

Structural transformation and denaturation of proteins caused by heat processing may significantly affect protein digestion and absorption. According to previous studies, heating at temperatures sufficient to induce protein unfolding promoted the digestibility of soy and PPs under infant digestion conditions (Tang et al., 2022). Hall et al. (2022) investigated the effects of high-pressure processing and heat treatment on the digestibility of protein and starch in pea protein concentrate (PPC). They found that high pressure-treated PPC underwent greater proteolysis and exhibited distinct peptide patterns after static gastric digestion compared with untreated and heat-treated PPC. Previous studies on heat-treated pea protein (HPP) have shown different heating trends; however, these studies did not provide data on simulated *in vitro* digestibility of PP (Wang et al., 2012; Peng et al., 2016; Ma et al., 2011).

Therefore, this study sought to examine the gastric digestion behaviour of PP and HPP using an *in vitro* dynamic Human Gastric Simulator (HGS). Employing advanced dynamic *in vitro* digestion models offers an in-depth understanding of PP digestion in the stomach, including changes in its physicochemical properties and the evolution of its microstructure. This study aimed to elucidate the impact of different heat treatments on PP digestion, providing valuable reference data for food scientists and nutritionists to design food products with higher nutritional value and enhanced functional properties.

## 2 Materials and methods

### 2.1 Materials

Commercial PP was procured from Roquette Company (Clinton, IA, USA). According to the packaging, its nutritional composition included 80 % (w/w) protein (conversion factor 6.25), a maximum of 10 % (w/w) ash and a maximum of 10 % (w/w) crude fibre content. Water was purified using a Milli-Q apparatus (Millipore Corporation, Bedford, MA, USA) and was utilised for all experiments. All chemicals used were of analytical grade and were sourced from Sigma Chemical Company Ltd. (St. Louis, MO, USA) or BDH Chemicals (BDH Ltd., Poole, UK) unless otherwise specified.

### 2.2 Preparation of HPP samples

A 200 g protein dispersion with a concentration of 3.0 % (w/w) was prepared by dissolving PP powder in Milli-Q water. The PP samples were adjusted to pH levels of 7.8, 6.8, 6.5, 6.0 and 5.5 using 4 mol L^−1^ HCl and NaOH solution. Subsequently, the PP dispersion was heated at temperatures of 60 °C, 70 °C, 80 °C, 90 °C and 95 °C for 20 min. In other cases, the PP dispersion was heated at 90 °C for durations of 5, 10, 20, 30 and 40 min. Untreated PP was designated as the control, while heat-treated PP was referred to as HPP.

### 2.3 Protein solubility

Protein solubility was determined following the method reported by Lan et al. (2018), based on the Bradford assay with modifications (Bradford, 1976). Bovine serum albumin was selected as the standard protein to generate a standard curve. Coomassie Blue G250 solution was combined with 100 mL of 85 % phosphoric acid and adjusted to a final volume of 1 L with distilled water. The prepared solution was filtered to obtain the assay reagent (The solution was centrifuged at 4000×*g* for 10 min). The diluted protein supernatants (25-fold, 100 μL) were pipetted into test tubes, followed by the gradual addition of 5 mL of assay solution to each tube. Protein concentration was quantified using a UV/Vis spectrophotometer (UV-1600PC, VWR, Belgium) at a wavelength of 595 nm. Protein solubility was determined using Eq. (1).

(1) $$\text{Protein}\;\text{solubility}\;\left(\%\right)=\frac{\text{Protein}\;\text{concentration}\;\text{in}\;\text{supernatant}}{\text{Initial}\;\text{protein}\;\text{concentration}}\times 100\text{.}$$

### 2.4 Measurement of turbidity

A spectrophotometer (Synergy 2, BioTek Corporation, USA) was used to measure the turbidity of the PP dispersion. Distilled water served as the reference blank. Turbidity was quantified based on the absorbance of each sample at 600 nm (Jiang et al., 2022). To ensure consistency, the suspensions were thoroughly vortexed for 10 s before each turbidity measurement.

### 2.5 Particle size measurement

Following the method described by Li et al. (2023), particle size and size distribution were measured using a laser diffraction instrument (Mastersizer, 2000; Malvern Instruments, Malvern, Worcestershire, UK). The refractive indices for the dispersion medium and the protein particles were 1.33 and 1.45, respectively. The particle size of PP/HPP particles was characterised using the volume-weighted average diameter $\left[d_{4,3}\left(=\Sigma \frac{n_{i}d_{i}^{4}}{n_{i}d_{i}^{3}}\right)\right]$, where n_i_ represents the number of particles with diameter d_i_. All measurements were conducted in triplicate.

### 2.6 Protein surface hydrophobicity

Surface hydrophobicity of the samples was measured following the method described by Li et al. (2020). The ANS solution (8 mmol L^−1^) and HPP samples, ranging from 1 to 5 mg mL^−1^, were prepared using 10 mmol L^−1^ pH 7.0 phosphate buffer solution (PBS). Subsequently, 20 μL of ANS was added to 4 mL of each PP solution and incubated in a dark room for 15 min at 20 °C. Fluorescence intensity was then measured with a fluorescence spectrophotometer (LS55, PerkinElmer, USA) at an excitation wavelength of 371 nm and an emission wavelength of 467 nm.

### 2.7 Determination of free sulfhydryl group content

The free sulfhydryl group content of PP was measured following the method described by Jiang et al. (2022). The samples were diluted to a concentration of 2 mg mL^−1^ using a pH 8.0 Tris–Gly buffer (0.086 M Tris, 0.09 M Gly and 4 mM EDTA). Subsequently, 3 mL of the diluted samples were mixed with 30 mL of Ellman reagent (4 mg DTNB mL^−1^ in Tris–Gly buffer) and allowed to react in the dark for 15 min at room temperature. Buffer and DTNB served as the reagent blank, while HPP samples lacking DTNB acted as the protein blank. The absorbance was then measured at 412 nm using a spectrophotometer (BioTek Corporation, America Synergy 2). Calculations were performed as follows:

$$\mu \text{molSH}g^{-1}=\left(73.53\times A_{412}\times D/C\right)\text{,}$$

where 73.53 = 106/1.36 × 10^4^, 1.36 × 10^4^ is the molar extinction coefficient, D represents the dilution factor of the sample and C is the original protein concentration.

### 2.8 Determination of intrinsic fluorescence

The samples were diluted to a concentration of 0.5 mg mL^−1^ using 10 mmol L^−1^ pH 7.0 PBS. To minimise tyrosine interference, the Als intrinsic fluorescence spectrum was recorded at an excitation wavelength of 290 nm. The emission spectrum was then captured over a scanning range of 300–420 nm at a speed of 240 nm min^−1^ with a slit width of 5 nm, as outlined by Li et al. (2023).

### 2.9 Fourier transform infrared spectroscopy

Fourier transform infrared spectroscopy (FTIR) was performed using an FTIR-iD7 spectrometer from ThermoFisher (ThermoFisher Scientific, Franklin, MA, USA), equipped with an attenuated total reflection accessory (MIRacle Single Reflection, Pike Technologies, Madison, WI, USA). The dried samples were milled with a mortar and pestle, and the same sample quantity was loaded into the measuring cell. A tight pressure clamp with a flat tip ensured optimal contact between the sample and the equipment. The spectra were recorded against an air background between 4000 and 400 cm^−1^ with an average of 32 scans and a resolution of 4 cm^−1^. The data were smoothed using the Savitzky–Golay function, the baseline was corrected, and the data were normalised with Origin Pro (OriginLab, Northampton, MA, USA). Fourier self-deconvolution and second derivative analysis were performed, followed by peak fitting in the amide I region (1700–1600 cm^−1^). The amide I region is most responsive to the secondary structure of the protein (Barth, 2007). Gaussian peaks were assigned to their corresponding structures based on their centres. Each sample was measured in triplicate using freshly milled material.

### 2.10 Sodium dodecyl sulphate polyacrylamide gel electrophoresis

The samples were examined through electrophoresis, following the procedure outlined by Li et al. (2022). Protein denaturation due to heating was evaluated by analysing the protein composition under different temperatures and durations using sodium dodecyl sulphate polyacrylamide gel electrophoresis (SDS-PAGE) under non-reducing and reducing conditions. Additionally, protein hydrolysis by pepsin over time was assessed in initial and digested PPI samples within the HGS (i.e., gastric chyme) by analysing protein composition at various digestion times using SDS-PAGE under reducing conditions. The acrylamide gels used consisted of a 10 % separating gel, a 5 % stacking gel and 0.1 % SDS. Protein samples (1.0 %, w/v) were mixed with a buffer containing 0.0625 M Tris–HCl, 10 % glycerin, 10 % SDS, 5 % 2-mercaptoethanol (βME) and 0.0025 % bromophenol blue. The mixture was heated at 95 °C for 5 min in a boiling water bath and then cooled to room temperature before being loaded into the gels. Electrophoresis was conducted at 80 mV for the stacking gel, followed by 120 mV for the separating gel until the tracking dye reached the bottom of the gel. The gels were subsequently destained using a solution of 10 % (v/v) acetic acid and 10 % (v/v) isopropanol and scanned with a Molecular Imager Gel Doc XR system (Bio-Rad Laboratories, Hercules, CA, USA). Precision Plus protein unstained standards (Bio-Rad Laboratories) were used to estimate molecular weights. Protein composition from the SDS-PAGE gels was quantified by densitometry using three replicates and analysed with Image Lab™ software version 5.2 (Bio-Rad Laboratories).

### 2.11 Confocal laser scanning microscopy

In accordance with the methodology described by Wang et al., 2022a, Wang et al., 2022b, the microstructures of PP/HPP, gastric chyme samples and digesta collected from the HGS were analysed using a confocal laser scanning microscope (Leica SP5 DM6000B, Leica Microsystems, Heidelberg, Germany). The samples were immediately stained and observed upon collection. Fast Green FCF (1.0 %, w/v) was employed to stain the protein (He–Ne laser with excitation at 633 nm). A 200 μL sample of PP or HPP was transferred into Eppendorf tubes and gently mixed with 5 μL of 1.0 % (w/v) Fast Green. Observations were conducted at least 5 min after dye diffusion into the samples. These samples were then placed on concave confocal microscope slides (Sail; Sailing Medical-Lab Industries Co., Ltd., Suzhou, China), covered with cover slips and observed using 20 × lenses (Leica HCX PL APO lambda blue 20 × 1.4 UV, Wetzlar, Germany). Images were captured at a resolution of 1024 × 1024 pixels using the microscope software (Leica).

### 2.12 Dynamic gastric digestion model and dilution effects

The digestion study was performed on heated (90 °C, 20 min) and unheated PP under pH 7.8 conditions. A dynamic HGS, developed by Kong and Singh (2010), was employed for the gastric phase of *in vitro* digestion. The simulated gastric fluid (SGF, pH 1.5) was prepared according to the method described by Wang et al., 2022a, Wang et al., 2022b, with slight adjustments. The SGF mixture contained freshly prepared solutions of KCl (6.9 mmol L^−1^), KH_2_PO_4_ (0.9 mmol L^−1^), NaHCO_3_ (25 mmol L^−1^), NaCl (47.2 mmol L^−1^), MgCl_2_·6H_2_O (0.1 mmol L^−1^) and (NH_4_)_2_CO_3_ (0.5 mmol L^−1^), dissolved in deionised water and stirred for 30 min to reach a final volume of 1 L (1.25 × concentration). Pepsin (4.8 g L^−1^), CaCl_2_ (0.15 mmol L^−1^) and additional water were added to adjust the electrolyte concentration, with pepsin and CaCl_2_ being introduced just before use. The pH of the SGF was adjusted to 1.5 using 1 M HCl/NaOH.

The HGS was preheated and kept at 37 ± 0.5 °C with an internal heater and thermostat. In each experiment, a 200 mL sample of the prepared PP and HPP solution was warmed to 37 °C and then transferred into the latex stomach chamber. To replicate gastric secretion, SGF–HCl and pepsin were introduced into the latex chamber at flow rates of 2.0 and 0.5 mL min^−1^, respectively. Gastric emptying was simulated by sampling gastric digesta at 20-min intervals through a silicone tube attached to the chamber bottom. A 50 mL digesta sample was filtered through a 1-mm pore mesh to simulate gastric sieving, retaining larger particles (Meyer et al., 1981). The filtered sample was designated as emptied digesta (Lobo et al., 2009), while any solids were returned to the stomach chamber. The HGS replicated natural gastric contractions at a frequency of three contractions per minute (Marciani et al., 2001).

Due to the gradual addition of SGF, samples were continuously diluted as the digestion process progressed. Assuming the sample was distributed homogeneously in the stomach throughout the entire digestion process, the resulting concentration after dilution was calculated; the calculation and the dilution line are shown in Fig. S1 (Supporting Information).

### 2.13 Quantification of nitrogen transfer after *in vitro* digestion

Chemical analyses, including dry matter and total nitrogen contents, were performed on the undigested PP/HPP and the digesta that was emptied from the bottom of the HGS during gastric emptying. Pepsin hydrolysis in the emptied digesta was stopped by adjusting the pH to 7.5 using 10 M NaOH. Dry matter content was determined via the forced-air oven method, where samples were placed in dry aluminium dishes and dried at 105 °C until a constant weight was achieved. The total nitrogen contents of the initial PP and the emptied digesta during the simulated gastric digestion were determined by a Kjeldahl method according to AOAC International (2006). A conversion factor of 6.25 (Mondor et al., 2020) was applied to PP to obtain the protein content from the total nitrogen content.

### 2.14 pH measurement

The initial pH, representing the pH of PP/HPP before digestion, was recorded using a pH meter before the digestion process commenced. Due to the contractions of the latex stomach bag in the HGS, direct access was restricted. Therefore, to obtain pH measurements at various time points during gastric digestion, digestion experiments were concluded at specific intervals. The collected gastric chyme was then thoroughly mixed using a stirring bar, and the gastric pH was measured. Each pH measurement was performed three times to ensure accuracy.

### 2.15 Physical stability

Following the method outlined by Wang et al. (2020), experiments were concluded at specific intervals to assess the physical state and stability of PP/HPP during digestion, as direct observation of gastric chyme within the HGS was obstructed by the latex stomach bag. Gastric chyme samples were retrieved from the stomach chamber. To halt the pepsin digestion, an aliquot of pepstatin A (0.5 mg mL^−1^ in methanol) was introduced into the gastric chyme (final concentration of 10 μL mL^−1^). The samples were gently mixed for a few seconds, and a 4 mL aliquot was then transferred to a glass tube sealed with a plastic cap. To monitor the instability due to sedimentation, photographs were taken at 0-, 20-, 40-, 60-, 120-, 180- and 240-min intervals after mixing, while the samples were held at room temperature using a digital camera. Initial changes during storage (0, 10, 30, 60 and 120 min) provided direct insight into the appearance of gastric chyme in the stomach.

### 2.16 Statistical analysis

Each experiment was conducted at least three times with freshly prepared samples. Results are presented as means and standard deviations. Statistical analysis was performed using one-way analysis of variance with the SPSS 19.0 software package (IBM, Armonk, NY, USA). Duncan's multiple range test was employed to identify significant differences among mean values, with a significance level set at *p* < 0.05.

## 3 Results and discussion

### 3.1 Effect of heat treatment on physicochemical properties of PPs

**Fig. 1:**
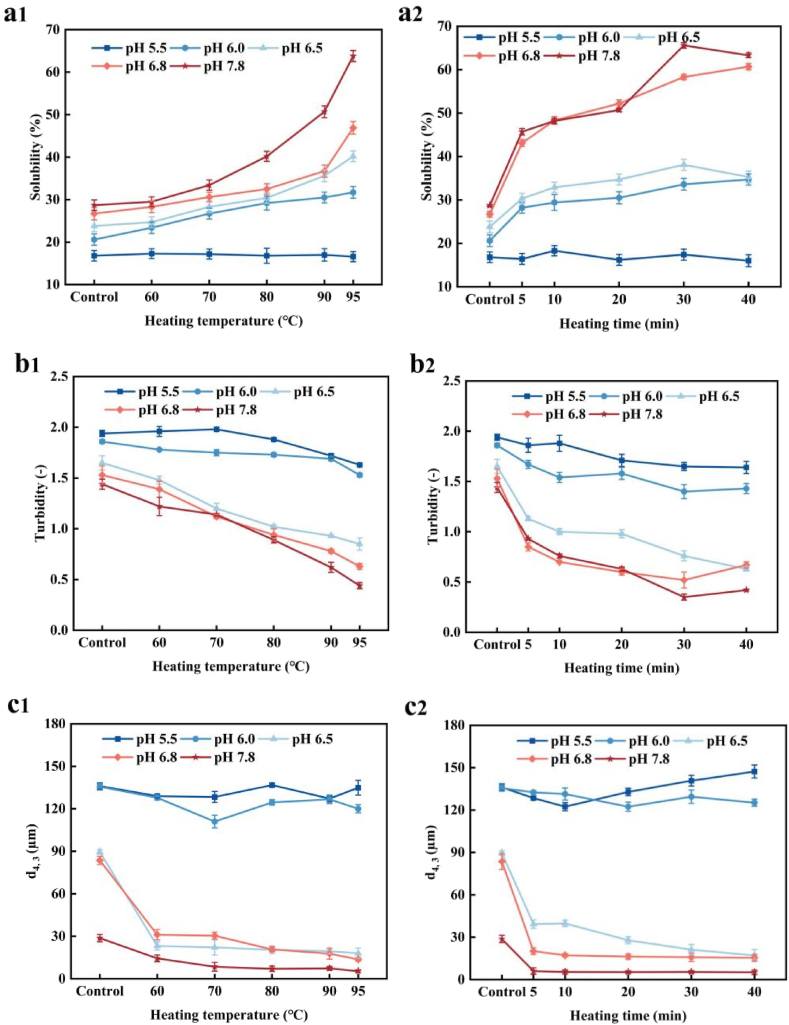
The solubility (**a1-a2**), turbidity (**b1-b2**) and particle size (**c1-c2**) of heat-treated PP at different pH. Samples (a1, b1 and c1) were heated at different temperature for 20 min. Samples (a2, b2 and c2) were heated at 90 °C for different time.

**Fig. 2:**
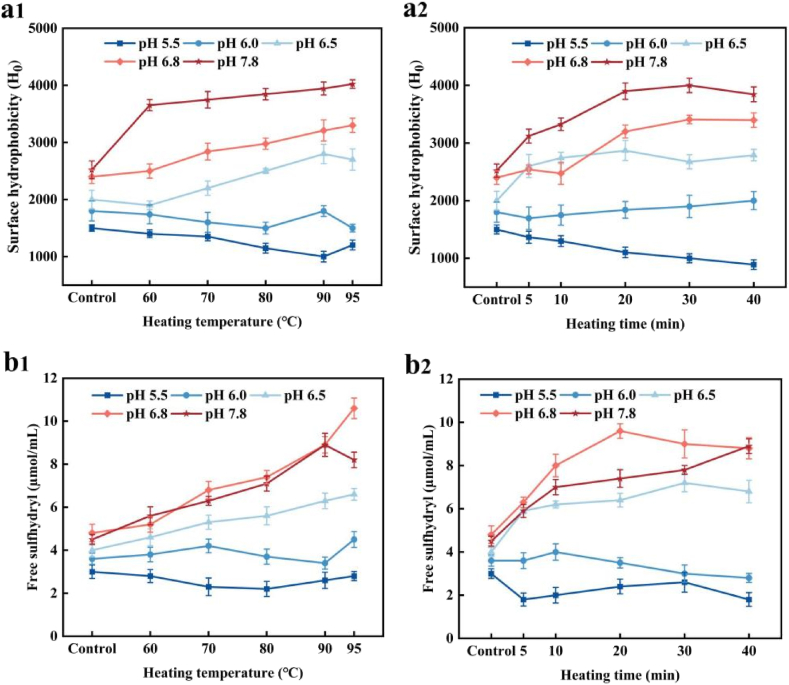
Surface hydrophobicity (**a1-a2**) and free sulfhydryl group (**b1-b2**) of PP with different heat treatment at different pH. Samples (a1, b1) were heated at different temperature for 20 min. Samples (a2, b2) were heated at 90 °C for different time.

Fig. 1 shows the solubility, turbidity and average particle size (d_4,3_) of PP heated at various temperatures and pH levels. The solubility of the untreated protein showed a significant pH dependency (Fig. 1, Fig. 2), decreasing as the pH decreased. Similar behaviour has been extensively documented for various plant-derived proteins (Barac et al., 2018) and is attributed to the formation of insoluble protein aggregates due to reduced electrostatic repulsion as pH decreases. At pH ≥ 6.0, PP solubility significantly increased with rising temperature, reaching its highest value (63.8 ± 0.31 %) after heating at 95 °C for 20 min at pH 7.8. At low pH (5.5), PP solubility is minimally affected by heat treatment, remaining nearly unchanged as the heating temperature increases. The lowest solubility (16.8 ± 0.23 %) was observed at 95 °C for 20 min at pH 5.5, where the pH is near the isoelectric point. This proximity weakens electrostatic repulsion between protein molecules, promoting aggregation and thereby reducing solubility (Dumetz et al., 2008). As shown in Fig. 1(a2), PP solubility increased with heating time at 95 °C at pH 7.8 and 6.8. At pH 6.5 and 6.0, although solubility continued to rise with heating time, the increasing trend was less pronounced than at pH 6.8. At pH 5.5, prolonged heating had no effect on solubility.

Turbidity is a vital indicator for evaluating the stability and solubility of protein dispersion. As shown in Fig. 1(b1–b2), the control sample was relatively transparent, with low turbidity (1.44 at pH 7.8), which decreased as the pH increased. This result was consistent with previous findings (Jiang et al., 2017). Within the pH range of 6.5–7.8, the turbidity of heat-treated samples decreased as the heating temperature and duration increased. The turbidity of the control samples in the pH range of 6.0 and 5.5 was >1.8, suggesting the formation of larger aggregates under these conditions.

As shown in Fig. 1(c1–c2), post heat treatment at different temperatures and durations, the d_4,3_ value of PP dispersion at pH ≥ 6.5 decreased by approximately 50 %–75 % as the heating temperature increased from 65 °C to 95 °C and the heating duration was extended. The lowest value (5.412 nm) was observed at 95 °C and pH 7.8. However, at pH 6.0, heating had minimal effect on PP particle size (d_4,3_). At pH 5.5, d_4,3_ slightly increased with rising temperature, likely due to greater protein particle aggregation at lower pH levels, resulting in larger and more irregular particle sizes. In contrast, while this study found that PP particle size decreased significantly with increasing heating temperature and time at pH > 6.5, Tang et al. (2022) reported an increase in PPI particle size during heat treatment. This discrepancy may be attributed to several factors. First, differences in protein source and purity between the two studies could affect aggregation tendencies. Second, variations in heating protocols, such as heating rate, holding time, and temperature control, can lead to distinct protein denaturation and aggregation pathways. Third, differences in sample preparation, including protein concentration and buffer composition, may also influence protein interactions and size changes.

### 3.2 Effect of heat treatment on structural characteristics of PP

Hydrophobic interactions in proteins are essential forces that maintain tertiary structure and influence protein stability, functionality and conformation. Surface hydrophobicity (H_0_) is an essential indicator for measuring the hydrophobic groups on the protein surface and has a greater impact on the functional properties of proteins than overall hydrophobicity (Chandrapala et al., 2011). Fig. 2 shows the surface hydrophobicity and free sulfhydryl group of PP before and after heat treatment at different pH levels.

The initial H_0_ value of PP was 2520 at pH 7.8 (Fig. 2, a1). As the pH decreased, the H_0_ gradually decreased to 1500 at pH 5.5. At pH ≥ 6.5, the H_0_ value of PP increased with increase in heating temperature and duration. This result aligns with previous studies: Yu et al. (2021) reported a similar increase in H_0_ with increasing temperature for egg white protein at pH 12, while Wang et al. (2012) found that heat-treated soy proteins exhibited higher H_0_ than that of unheated proteins. During heat treatment, the internal hydrophobic groups were gradually exposed, increasing the polarity of their environment. At pH ≤ 6.0, H_0_ decreased as heating temperature increased, reaching its lowest value (1000 at pH 5.5) after heating. This suggests that fewer hydrophobic groups were exposed, resulting in a more compact structure. Under acidic conditions, unfolded proteins tend to aggregate, causing exposed hydrophobic regions to re-aggregate. This re-aggregation limits ANS interaction with H_0_ groups, leading to a decrease in H_0_.

As shown in Fig. 2b, the free SH content generally increased with rising heating temperature and duration from control PP at pH ≥ 6.5. At pH ≤ 6.0, however, free SH content decreased with heat treatment, probably due to protein aggregation. The increase in free SH content was closely related to conformational changes and protein unfolding, indicating the exposure of sulphydryl groups or the disruption of disulphide bonds (Nguyen, Wong, Havea, Guyomarc'H, & Anema, 2013). This shows that tertiary structure loss in PP became more pronounced with prolonged heating. Additionally, free SH content in PP treated at pH ≤ 6.0 was lower than that at pH ≥ 6.5, suggesting that PP structure was more significantly affected at lower pH. The SH value decreased with extended heating due to disulphide bond formation.

Results indicate that different heat treatment conditions have varying effects on the free SH content and protein structure of PP. During heat treatment, proteins undergo structural and conformational changes, including unfolding (denaturation), exposure of hidden reactive groups such as sulfhydryl (–SH) and cysteine amino acids, protein–protein interactions via hydrophobic forces and the formation of new disulphide linkages through reactions between the –SH side chains of two cysteine residues. The presence of SH has been shown to enhance protein solubility, a key functionality that supports the utilisation of proteins as food ingredients (Mundi et al., 2012; Yildiz et al., 2018). Exposed SH groups in a protein can serve as an indicator of conformational changes, such as structural unfolding or the cleavage of –S–S– bonds during processing. Studies by Wu & Kuwajima et al. (2022; 1989) indicate that a reduction in free sulfhydryl content in PP could signify that heat treatment facilitates the formation of disulphide bonds between polypeptide chains at pH 7–8.

At an excitation wavelength of 290 nm, the obtained fluorescence spectra primarily reflect the emission from tryptophan residues. Changes in the tertiary structure and side chains of the protein after pH-induced heating were elucidated. The *λ_max_* of PP subjected to heat treatment at different pH levels is presented in Table 1. Heat treatment has a significant impact on the shape of the fluorescence spectra of PP, with the *λ_max_* value and fluorescence intensity undergoing varying degrees of change (Table 1). Results indicate that all *λ_max_* values are >330 nm, suggesting that the Trp residues are located in a polar environment on the exterior of the protein molecules (Vivian et al., 2001). Compared with untreated protein samples, the *λ_max_* values of PP samples under different heat treatments varied. When pH ≥ 6.5 and heat treatment was <90 °C for 40 min, a slight red shift in the maximum fluorescence wavelength of Trp was observed, indicating that PP or its subunits underwent minor aggregation changes. At pH ≤ 6.0, the *λ_max_* value blue-shifted from 347 to 340.5 nm, indicating changes in the protein structure. This change is due to the disruption of hydrophobic interactions between protein molecules by heat treatment, causing the side chains of tryptophan residues to become gradually exposed on the molecular surface, increasing the polarity of their environment and resulting in a blue shift of the maximum fluorescence wavelength (Liu et al., 2015). When heat treatment was applied at 90 °C for more than 20 min, the degree of blue shift in the maximum fluorescence wavelength of Trp decreased. This suggests that, over time, the PP structure gradually transitioned from a loose to a more compact state. As shown in Fig. S2 (Supporting Information), the intrinsic fluorescence intensity of HPP at pH ≥ 6.5 is stronger than that of PP and increases with prolonged heating time at 90 °C. This phenomenon suggests that under neutral pH conditions, prolonged heating induces the extension and unfolding of the internal structure of the protein. In this state, tryptophan residues become exposed on the protein surface, leading to changes in fluorescence intensity. The degree of tryptophan residue exposure increased with extended heating time. The heat treatment at neutral pH enhanced the repulsive forces in PP, thereby exposing more hydrophobic groups (Jiang et al., 2011).

**Table 1:** The *λ_max_* of PP treated with heat treatment at different pH.

Sample	*λ_max_* (nm) pH 7.8	*λ_max_* (nm) pH 6.8	*λ_max_* (nm) pH 6.5	*λ_max_* (nm) pH 6.0	*λ_max_* (nm) pH 5.5
Control	340.5	340	341.5	344.5	347
60 °C	341.5	342.5	344	341.5	342
70 °C	341	340.5	342.5	343	341
80 °C	342	340.5	342.5	344.5	340.5
90 °C	344	342.5	341.5	343.5	339.5
95 °C	342	342.5	343	342.5	340.5
5 min	343	341	342.5	341	342
10 min	343	340.5	342.5	341.5	339
20 min	342.5	343.5	342	343.5	342.5
30 min	343.5	342	342.5	343.5	343.5
40 min	344	342	340.5	343	342.5

**Fig. 3:**
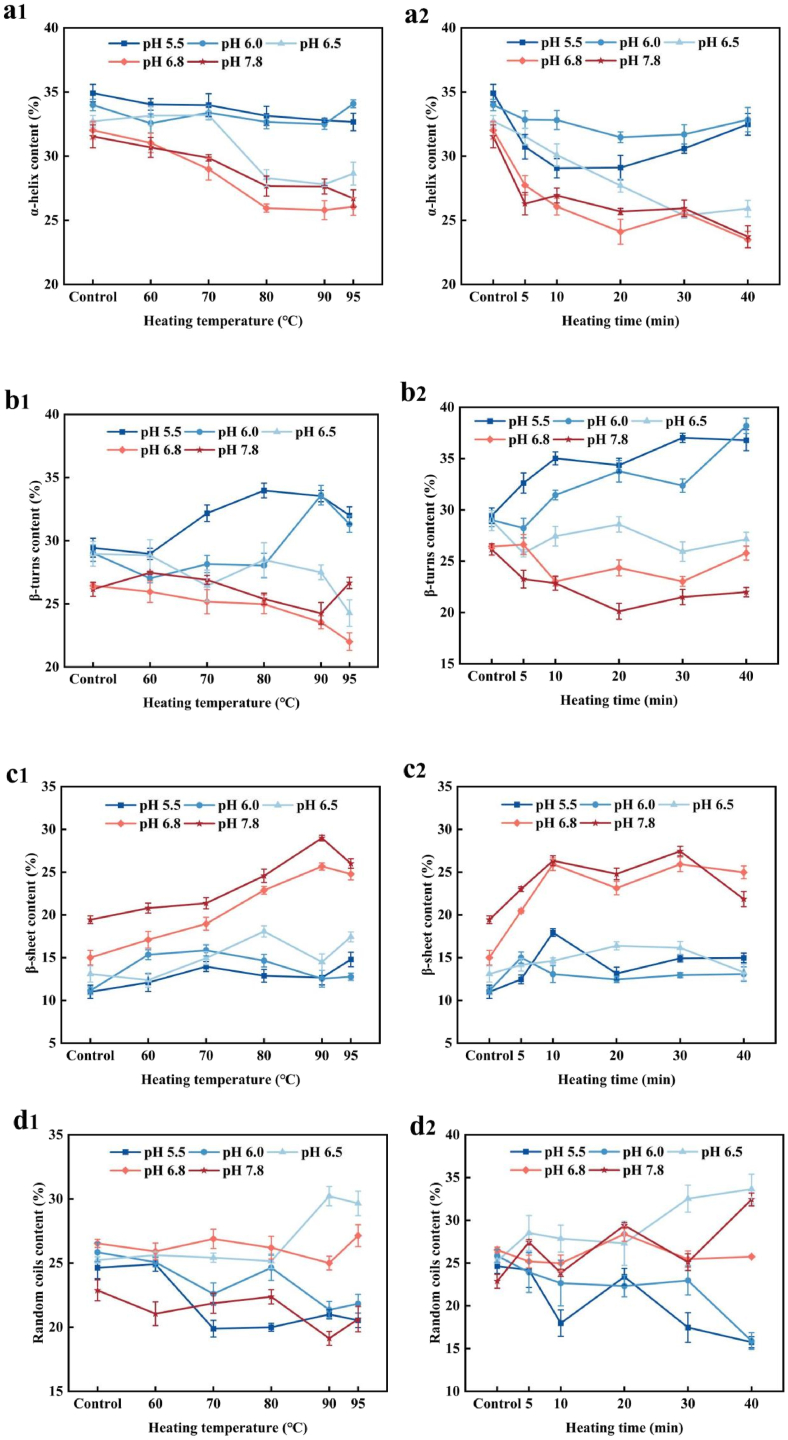
The relative content of secondary structure components of PP/HPP at different pH. Samples (a1, b1, c1, and d1) were heated at different temperature for 20 min. Samples (a2, b2, c2 and d2) were heated at 90 °C for different time.

In summary, as the heating temperature increased, the maximum fluorescence wavelength initially underwent a slight red shift followed by a minor blue shift, and the fluorescence intensity increased significantly after high-temperature treatment. Regarding pH, when the pH was lower, the maximum fluorescence wavelength exhibited a slight blue shift, and the fluorescence intensity was higher. This could be due to the refolding of the protein at low pH, under the same heating time and temperature conditions, indicating that the spatial conformation of PP underwent certain changes during heat treatment, which is consistent with the FTIR results (Fig. 3). These results suggest that a combination of neutral pH and heating effectively influences tryptophan distribution in PP, increasing its endogenous fluorescence intensity. Chao et al. (2018) reported that heat treatment above the denaturation temperature induces protein unfolding, exposing previously buried tryptophan residues to the hydrophilic environment. This demonstrates that heat treatment enhances the polarity of the aromatic amino acid environment, consequently strengthening the interaction between fluorescent chromophores and water molecules, leading to a red shift and intensified fluorescence intensity (Tao et al., 2019; Malik et al., 2019).

### 3.3 FTIR spectroscopy

Secondary structure changes in PP before and after heat treatment were characterised using FTIR measurements, which provided the protein molecular amide I band (1600–1700 cm^−1^). The amide I band infrared spectra of PP samples subjected to heat treatment at different pH levels were fitted using the Gaussian area method, and the second derivative was obtained. This yielded the fitted amide I band spectra of PP, from which the content of the protein secondary structure was calculated. Fig. 3(a1–d2) shows the calculated results for α-helix, β-turn, β-sheet and random coil structures.

As shown in Fig. 3a, b, the α-helix and β-turn content of heat-treated PP samples significantly decreased compared to PP at pH ≥ 6.5. However, this decrease was not pronounced at temperatures below 70 °C. This may be because the denaturation temperature of PP is above 74 °C, and heating at 60 °C did not affect its protein structure (Mession et al., 2015). At pH ≤ 6.0, the α-helix content showed no significant changes with increasing heating intensity. The reduction in α-helix and β-turn content suggests partial unfolding or loosening of the secondary protein structure, potentially exposing previously hidden hydrophobic amino acids on the protein surface (Chu et al., 2019).

As shown in Fig. 3c, d, at pH ≥ 6.5, the β-sheet content increased with rising heating temperature and duration. At 90 °C for 20 min, the proportion of β-sheet increased to 28.98 % at pH 7.8. However, the trend in random coil content was less pronounced than other secondary structures. As heating temperature and duration increased, the random coil content slightly increased. At pH ≤ 6.0, the random coil content decreased, indicating increased protein aggregation, which heat treatment was unable to mitigate.

In conclusion, heating significantly reduced the α-helix content while increasing the β-sheet content at pH ≥ 6.5, consistent with previous findings (Zhao et al., 2020). However, at pH < 6, heating did not noticeably alter the secondary conformation of PP. The differences in secondary structure may be attributed to an unfolding–refolding process induced by pH-shifting treatment. Heating could disrupt hydrogen bonds between polypeptides, leading to a decrease in PP molecular-weight and enhanced electrostatic repulsion at pH ≥ 6.5, which influences molecular unfolding and rearrangement (Wang et al., 2018).

### 3.4 SDS-PAGE

**Fig. 4:**
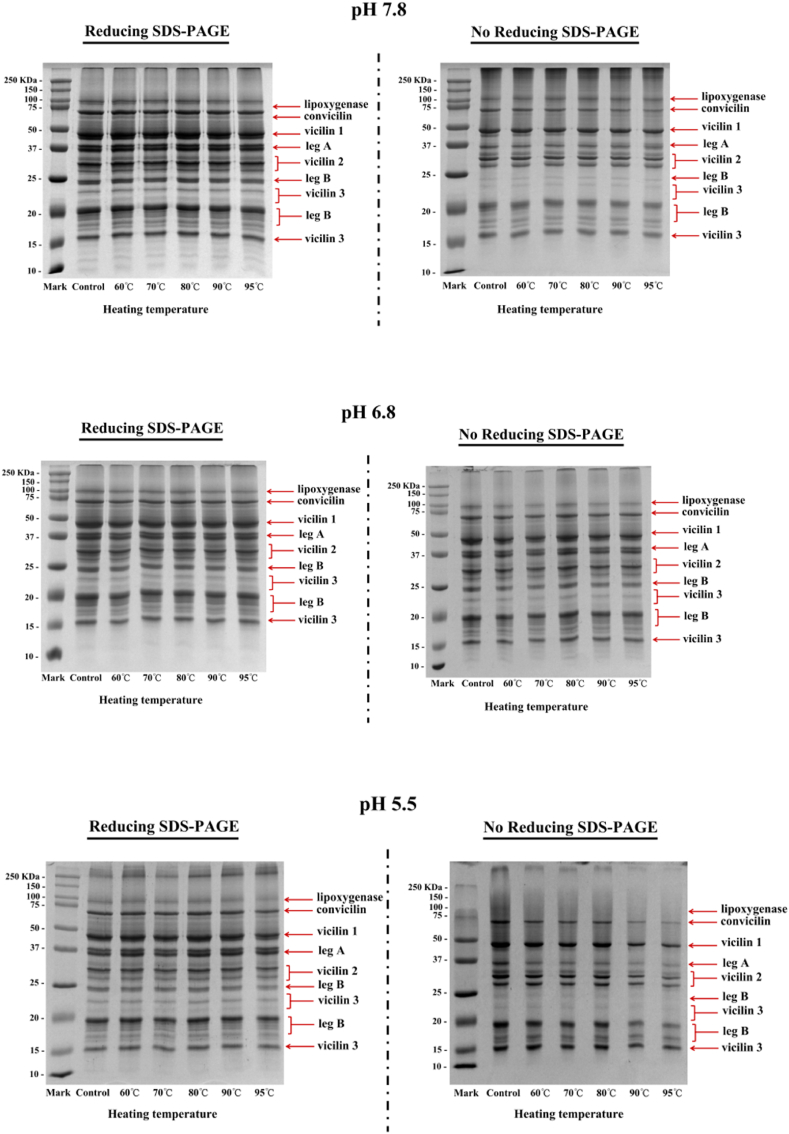
SDS-PAGE patterns under reducing (Left) and non-reducing (Right) conditions of different PP/HPP samples (heated at different temperature for 20 min) at different pH.

As shown in Fig. 4, unheated PP consisted of multiple components, primarily attributed to legumin and vicilin. The 75 kDa band was associated with convicilin, which is considered the α-subunit of vicilin due to its high degree of homology with vicilin (O'Kane, Happe, Vereijken, Gruppen, & Van Boekel, 2004). The bands of 50, 31–34 and 10–12 kDa could be attributed to vicilin. There are no disulphide bonds (SS) between different polypeptides of vicilin. Moreover, the subunits with the molecular weights of 38 and ∼20 kDa can be attributed, respectively, to the acidic subunit (leg A) and basic subunit (leg B) of legumin. Legumin has six subunit pairs, which consist of an acidic and a basic subunit linked by a single disulphide bond.

To gain insight into the differences in subunit composition after heating, SDS-PAGE was performed under reducing and non-reducing conditions (Fig. 4). As shown in the reducing gels in Fig. 4, the subunits remained distinct and unchanged across all samples, despite heating at different temperatures up to 95 °C. Tang et al. (2022) found that with increased heating intensity, soy and PPs unfolded without affecting the primary structure, which was manifested as increased surface hydrophobicity, thereby potentially improving accessibility to digestive enzymes.

The similarity between PP and HPP was that most of the subunits remained stable under non-reducing conditions, except for convicilin, even after heating at 95 °C. Regarding the convicilin protein subunits, Fig. 4 (right) shows that samples heated at 60°C–95 °C under different pH conditions exhibited strong intensity of intact convicilin subunit bands. However, after heating at 80 °C, the intensity of these bands diminished. Simultaneously, aggregates too large to migrate into the gel were visible in the wells at the top. The detection of soluble aggregates by SDS-PAGE has been previously reported (Chen et al., 2020).

Overall, SDS-PAGE results indicate that heat treatment under different pH conditions did not cause significant changes in any protein subunits, aligning with previous findings that heat treatment does not affect protein subunits (Laguna et al., 2017). Additionally, the molecular weight distribution of HPP was similar to that of PP and was not affected by acidic pH treatment. This suggests that the heat treatment or environmental stress (pH) applied to PP has a limited impact on the composition of protein particles. Moreover, results from the non-reducing gels demonstrate that a considerable portion of PP did not enter the separating gel but remained in the stacking gel, indicating that they are oligomers >250 kDa. This suggests that the heat treatment in this study might lead to the formation of the covalent disulphide bonds in PP.

### 3.5 Microstructure of protein particles

**Fig. 5:**
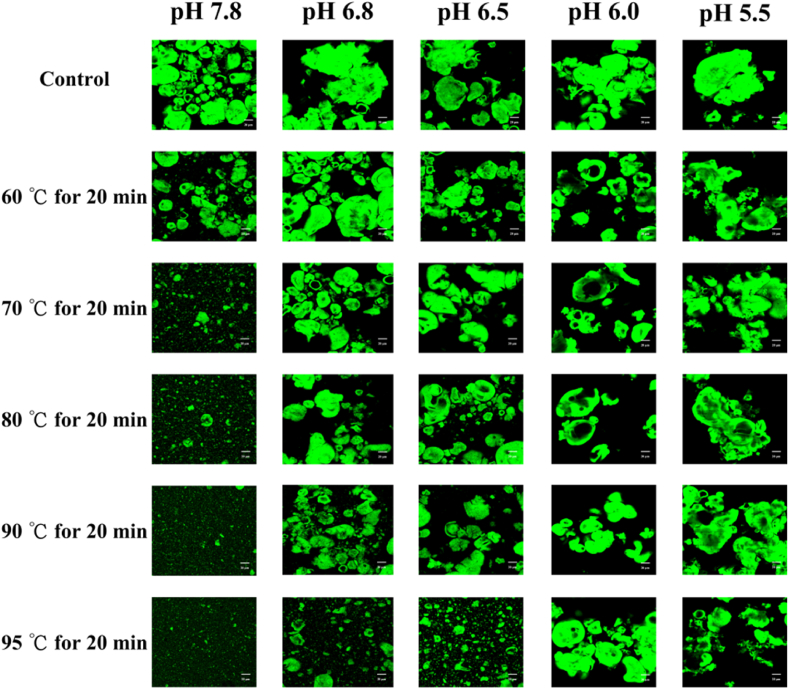
Confocal laser scanning microscopy (CLSM) images of the PP solutions after different heating temperature for 20 min at different pH (scale bar: 20 μm).

Fig. 5 shows representative microscopic images of the PP microstructure at different pH levels under varying heating temperatures and times. For untreated PP (the control), notable structural differences were observed across different pH values. As the pH decreased, the protein particle size of PP significantly increased. For heated samples, at pH ≥ 6.5, the protein particle size gradually decreased with increasing heating temperature and time, especially at pH 7.8. The smallest particle size was observed at 95 °C for 20 min (Fig. 5), aligning with results from particle size measurements (Fig. 1, Fig. 2). The lighter regions within the protein micron-scale aggregates may correspond to loosely packed zones, potentially associated with internal structural heterogeneity or retained water content during sample preparation. When pH ≤ 6.0, the particle size after heating showed no apparent change compared to the control, with protein particles being nearly identical. This indicates that under low pH conditions, heating has a minimal effect on the particle size of PP. By comparing the microstructure of the dispersions before and after heating, it is clear that under neutral pH conditions (pH ≥ 6.5), heating reduced the particle size and improved solubility of PP, resulting in smaller protein particle with compact protein micron-scale aggregates with relatively smooth contours and occasional surface depressions. Additionally, the particles in the heated PP solution appeared more uniform than those in the unheated protein solution. These findings are consistent with previous studies stating that thermal treatment results in fewer aggregates and a more homogenous protein dispersion (Ben-Harb et al., 2018).

### 3.6 Characterisation of physical changes and digestion behaviour during *in vitro* gastric digestion

#### 3.6.1 Changes in gastric chyme

**Fig. 6:**
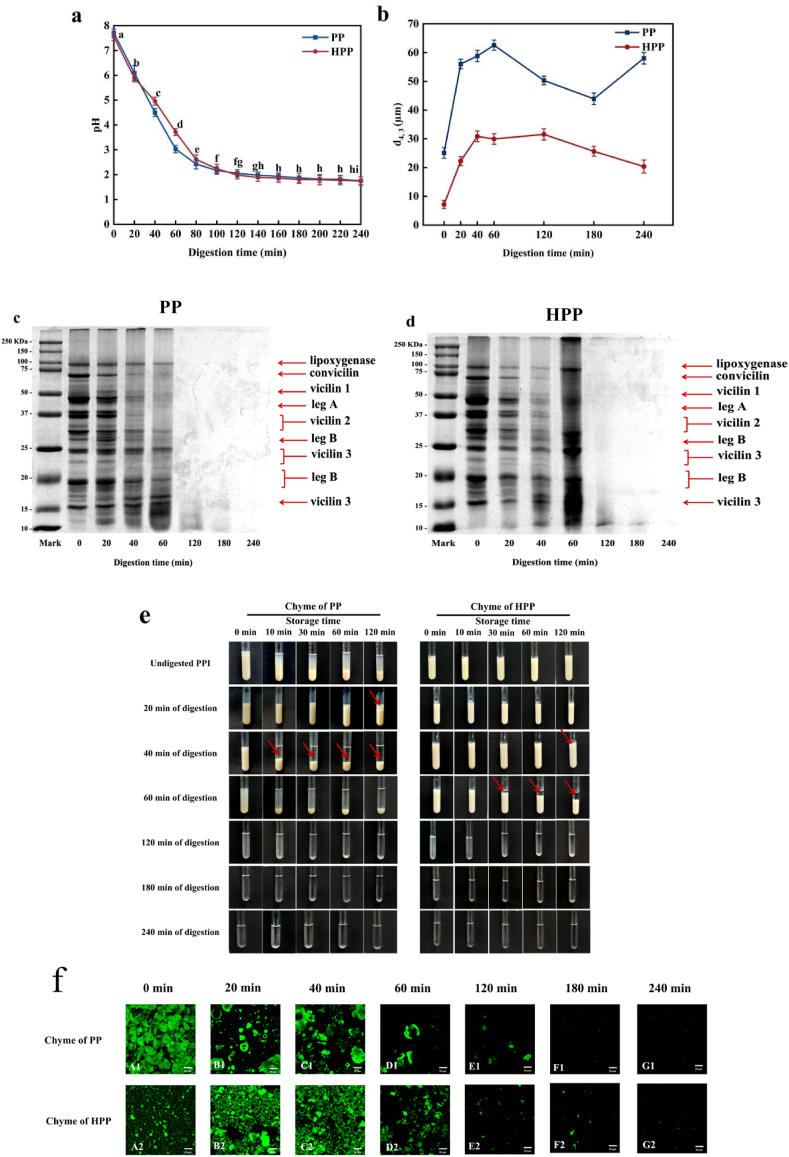
The pH (**a**), the average particle size (d_43_) (**b**), SDS-PAGE under reducing conditions **(c** and **d**), physical state (**e**), and confocal microimages (**f**) of PP/HPP samples (gastric chyme) during gastric digestion in the HGS.

Fig. 6 shows the changes in pH, particle size, SDS-PAGE results, physical state and confocal micrographs of gastric chyme samples (PP/HPP heated at 90 °C for 20 min at pH 7.8) during simulated gastric digestion. In general, the pH of all samples decreased over time, with a slight difference in pH profiles between heated and unheated PP. In unheated PP treated with SGF containing pepsin, the pH dropped sharply to around 2 within the first 100 min and then remained relatively stable throughout the remainder of digestion, up to 240 min (Fig. 6a).

The particle size varied during digestion in heated and unheated samples (Fig. 6b). Initially, the average volume-weighted mean diameter (d_4,3_) of PP was approximately 29.1 μm, while that of HPP was around 7.2 μm. The particle size increased during digestion up to 60 min and then gradually decreased until 240 min in PP and HPP, with HPP samples consistently exhibiting larger size values than PP samples. The initial increase in particle size may be attributed to PP protein aggregation due to pH reduction and the presence of pepsin, whereas the subsequent decrease is likely due to the dissociation of protein aggregates induced by pepsin-mediated hydrolysis at low pH.

As the gastric pH decreased from 4.5 at 60 min to 1.9 at 150 min, the protein matrix gradually dissociated due to pepsin-mediated protein hydrolysis and shear forces in the HGS (Wang et al., 2021). Wang et al. (2021, 2022) studied gastro-jejunal digestion of soymilk in HGS, observing that curd particles became smaller in size and were gradually emptied as gastric emptying progressed. Their findings indicated that the protein matrix dissociated over time as a result of pepsin hydrolysis and shear forces within the HGS.

Fig. 6c, d shows SDS-PAGE patterns of the protein profiles in gastric chyme. During gastric digestion by pepsin (Fig. 6c), all protein bands progressively decreased in intensity within the first 20 min. From 40 min onwards, numerous low molecular weight peptides (<15 kDa) became visible. By 60 min, the bands corresponding to major PP had nearly disappeared, leaving only low molecular weight peptides. After 120 min, almost all bands had vanished. Moreover, heat treatment enhanced proteolysis, showing more small peptides, especially after 60 min of digestion (Fig. 6d). The differences in protein hydrolysis rates between unheated and heated PP samples may be attributed to structural variations in the aggregates formed during heat treatment. The denser aggregation structure in unheated PP (Fig. 6f) slowed the rate of protein hydrolysis. This suggests that when large protein aggregates are formed, the diffusion of pepsin into the interior of these aggregates is hindered, potentially limiting proteolytic access. Moreover, the limited surface area of the aggregation restricts the rate of hydrolysis. In contrast, the aggregation formed in heated PP has a loose structure and a significantly larger surface area, facilitating the rapid diffusion of pepsin into the aggregate and thereby enhancing protein hydrolysis (Jiménez-Munoz et al., 2023; Yang et al., 2022).

Fig. 6e shows changes in the physical stability of PP and HPP samples during digestion. PP began to aggregate after approximately 20 min of digestion, with visible sedimentation observed at this stage (Fig. 6e, chyme of PP, 120 min). The gastric chyme separated into a white sediment layer at the bottom (indicated by red arrows in Fig. 6e, chyme of PP, 40 min) and a transparent serum layer at the top, with tiny white protein particles floating within the serum layer. As digestion progressed, the amount of sedimented and floating particles decreased, and after approximately 180 min, most of the sediment was discharged from the bottom of the HGS (Fig. 6e, chyme of PP, 30 min). In HPP samples, protein aggregation and coagulation were observed around 40 min into digestion (120 min), occurring later than in PP samples. Sedimentation became clearly visible after 60 min of digestion, with a substantial accumulation of protein particles at the bottom and a transparent serum phase at the top (Fig. 6e, chyme of HPP, 120 min). These particles were smaller and softer than those in the PP sample. As digestion progressed, the particles gradually decreased in size and were progressively emptied.

CLSM provided additional information on the microstructural changes in the PP inside the HGS during the simulated gastric digestion (Fig. 6f). Undigested PP is essentially a protein solution, with protein particles dispersed in the aqueous phase. The aggregation of PP after 40 min of digestion was evident in the microstructure of the gastric chyme, appearing as large clusters of protein particles. As digestion progressed, smaller protein aggregates became visible. By the end of simulated gastric digestion with pepsin, only tiny protein particles remained. During simulated gastric digestion, the particle size of HPP was significantly smaller than that of PP (Fig. 6f). This result further confirmed that neither PP nor heated PP formed a curd throughout the digestion process. In contrast, earlier studies on cow's milk (Roy et al., 2021; Ye, 2021 ) and soy milk (Wang et al., 2020, 2022) have shown that curd formation occurs during digestion due to the pepsin-induced coagulation of milk proteins.

#### 3.6.2 Changes in emptied gastric digesta

The gastric contents were emptied from the bottom of the HGS at various digestion times. The emptied digesta represented the material that progressed to the next stage of digestion (i.e., the intestinal phase).

**Fig. 7:**
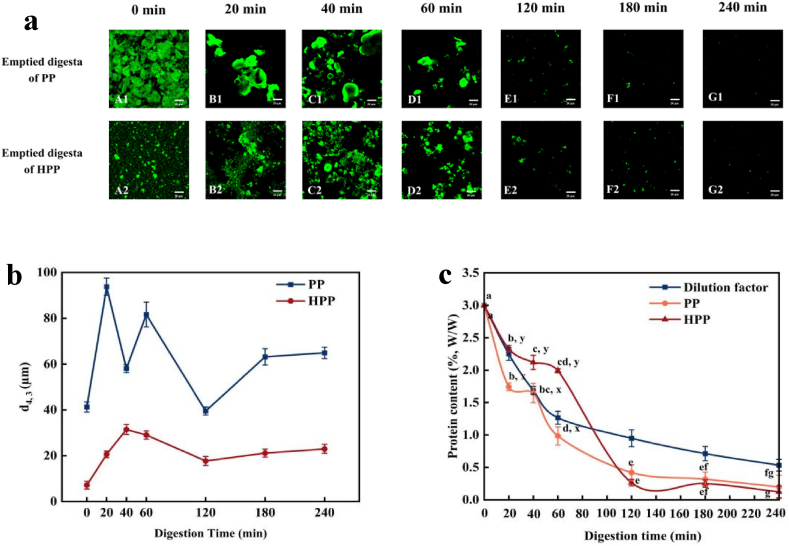
The average particle sizes (d_43_) (**a**), confocal microimages (**b**), protein contents (w/w, %) (**c**) of the emptied digesta obtained from PP and HPP during gastric digestion in the HGS.

Changes in the microstructure and the particle size of the emptied digesta obtained from PP and HPP samples during gastric digestion in the HGS as a function of digestion time are shown in Fig. 7a, b. Significant differences in the particle sizes of the emptied digesta from the PP and HPP samples could have potential implications for their subsequent digestion in the small intestine. The emptied gastric digesta consisted of many tiny aggregates with irregular shapes and a range of different sizes of PP and HPP. However, the particle size of HPP digesta was always smaller than that of PP digesta during the entire digestion process. This result is similar to that of gastric chyme (previous section), further confirming that PP and heated PP did not form a curd throughout the entire digestion process.

The protein content of the digesta emptied from the HGS at various time points during gastric digestion was analysed (Fig. 7c). The blue lines shown in Fig. 7c indicate the calculated protein concentrations in the emptied digesta at each time point. The calculation was based on the assumption that proteins were uniformly distributed in the PP solution during the entire digestion process, as shown in Fig. S1 (Supporting Information). The decrease in calculated values resulted from the dilution effect caused by gradual gastric emptying. The dry matter and total nitrogen concentrations shown in Fig. 7c were adjusted to account for gastric dilution and variations in initial dry matter and total nitrogen contents. The corrected dry matter and total nitrogen delivery were calculated as the difference between the measured values of the emptied digesta and the estimated dilution values. These values were expressed as a percentage of the difference relative to the initial dry matter and total nitrogen content of the sample before digestion (Fig. 7c). Using protein content as an example, the changes in protein levels in the emptied digesta during gastric digestion were significantly influenced by heat treatment, digestion time and their interaction (Fig. 7c). In the first 120 min, the protein content in the emptied digesta of the heated PP sample was significantly higher than that of the unheated PP sample, suggesting a faster rate of protein emptying in HPP. This result may be attributed to the smaller particle size and greater hydrolysis of heated protein by pepsin in the heated PP sample during dynamic digestion.

## 4 Conclusions

This study examined the effects of heat treatment under varying pH conditions on the structural characteristics, aggregation behaviour and gastric digestion of PP, aiming to elucidate the relationship between heating parameters and structural changes, as well as their impact on plant protein digestion. The findings revealed that heat treatment at neutral pH resulted in the formation of soluble aggregates with smaller particle sizes and a uniform size distribution than that of PP without heating. However, under pH < 6.5, heat treatment did not effectively improve PP solubility. Furthermore, HPP isolates exhibited faster disintegration and higher hydrolysis efficiency during *in vitro* gastric digestion without forming protein curds. These findings help to elucidate how heat treatment and pH influence the structural and digestive properties of pea proteins, which can inform formulation strategies in complex food matrices where protein solubility, aggregation behavior, and digestibility are critical. For example, in emulsion systems, protein solubility and surface activity are essential for the adsorption and the formation of stable emulsion. Similarly, in meat alternatives, the digestibility and structural behavior of proteins under simulated gastric conditions can affect satiety and nutrient bioavailability.

## Declarations of ethical statement

The authors have declared no studies in humans and animals.

## Author statement

Dan Li: Methodology, investigation, formal analysis, writing—original draft, visualisation, Alejandra Acevedo-Fani: writing—review & editing, Ying Ma: supervision, writing—review & editing, Weihong Lu: supervision, writing—review & editing, Harjinder Singh: writing—review & editing, Aiqian Ye: conceptualisation, supervision, funding acquisition, methodology, project administration, writing—review & editing

## Declaration of competing interest

The authors declare no conflicts of interest and are solely responsible for the content and writing of this paper.

## Data Availability

Data will be made available on request.
