# Supplementary material for: Heat-induced modifications of pea protein: Implications for solubility and digestion behaviour
Source: Curr Res Food Sci. 2025 Aug 16;11:101173. doi: 10.1016/j.crfs.2025.101173 (PMC12745828; doi:10.1016/j.crfs.2025.101173)
Supplement: Multimedia component 1 [file mmc1.docx]

# In vitro gastric digestion of pea protein using a human stomach simulator: effects of heat treatment

**Dan Li ^a, b^, Ying Ma ^c^, Alejandra Acevedo-Fani ^a^, Weihong Lu ^c^, Xuepeng Li ^b^, Harjinder Singh ^a^, Aiqian Ye ^a^***

^a^ Riddet Institute, Massey University, Private Bag 11 222, Palmerston North, 4442, New Zealand

^b^ School of Chemistry and Environmental Engineering, Liaoning University of Technology, Jinzhou, China, 121013, China

^c^ School of Chemistry and Chemical Engineering, Harbin Institute of Technology, Harbin, 150001, China

* Corresponding author: Aiqian Ye

mail addresses: a.m.ye@massey.ac.nz


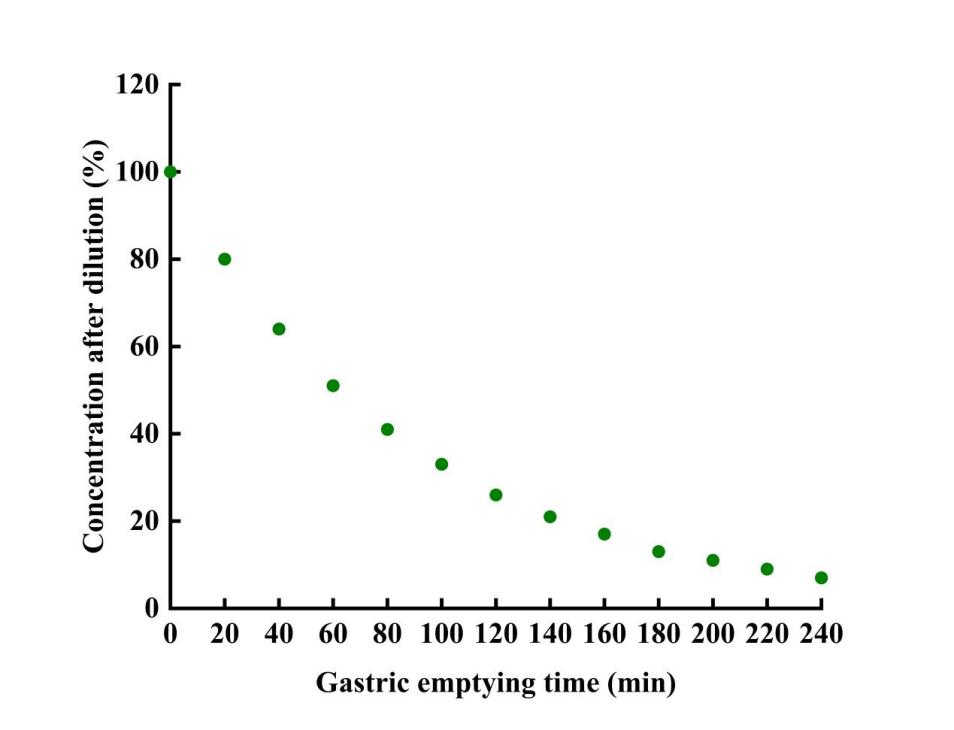


**Fig. S1.** The dilution line of sample.


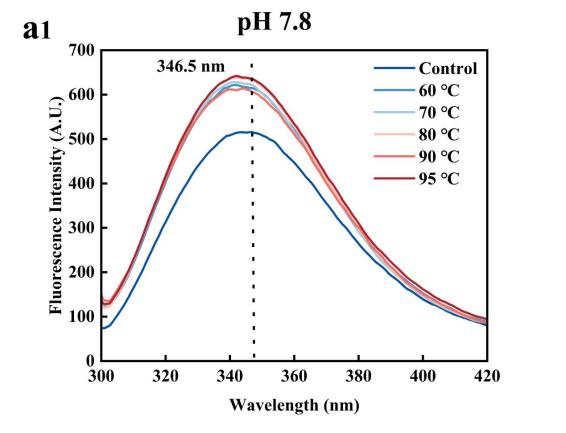

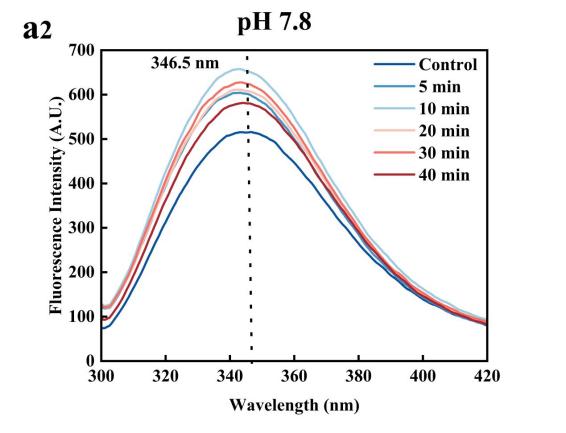

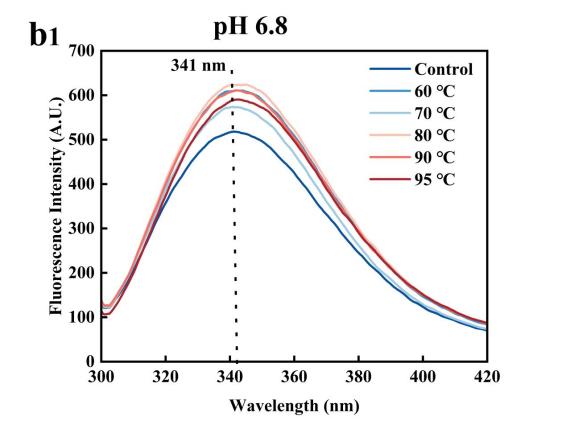

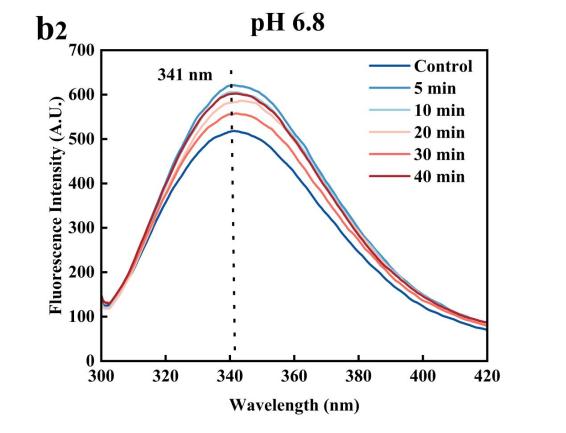

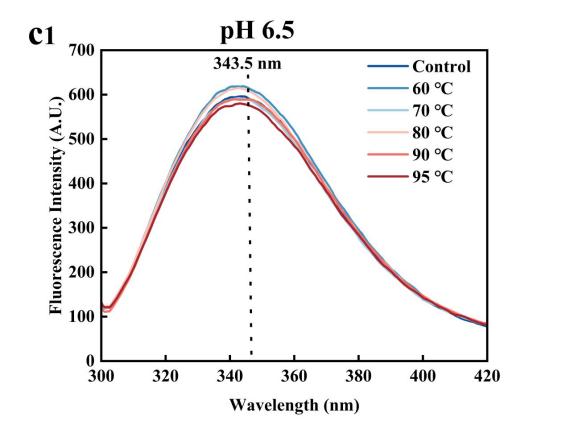

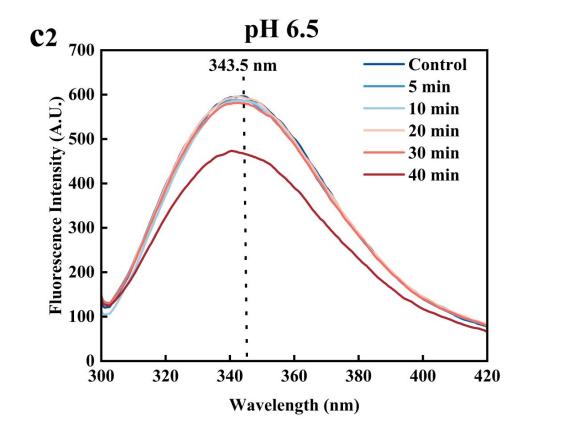

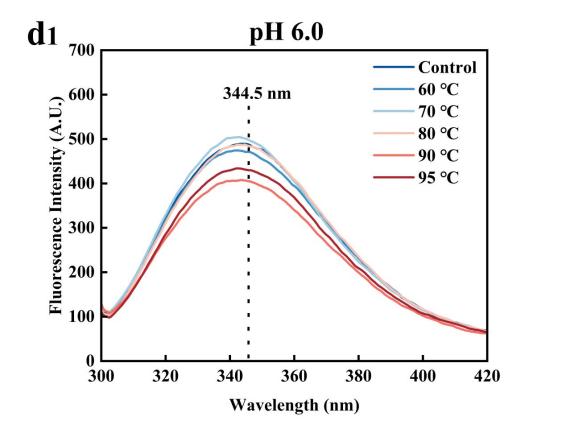

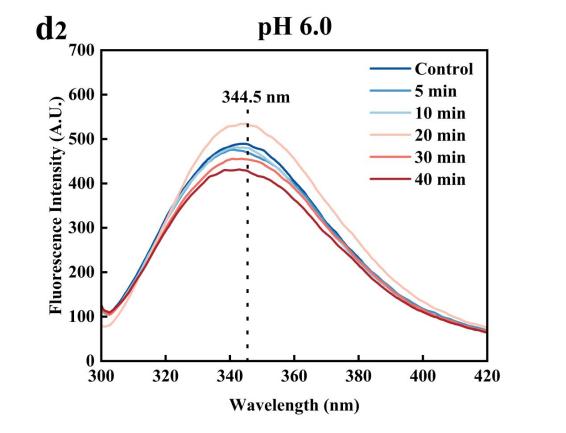

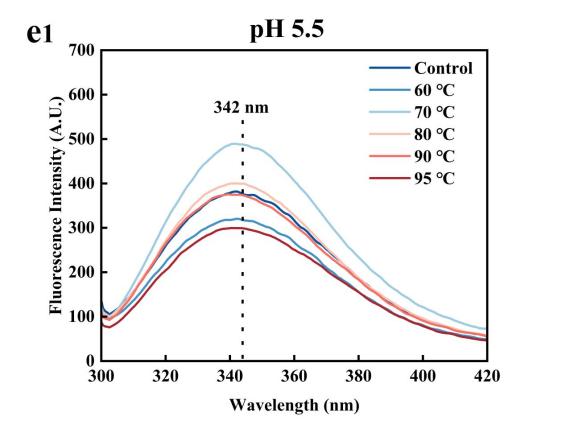

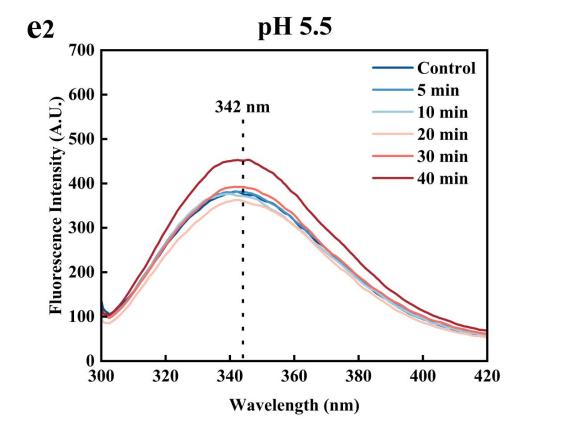


**Fig. S2.** Effect of heat treatment at different pH on fluorescence spectra of PP samples.
